# *Ab initio* quantum transport investigation of Sub-3 nm β-InSe transistors for future high-performance nanoelectronics

**DOI:** 10.1039/d5ra06179b

**Published:** 2025-10-13

**Authors:** Mughira Ghafoor, Rajwali Khan, Salah Ud Din, Akif Safeen, Quihui Li, Xingyue Yang, Zongmeng Yang, Jing Lu

**Affiliations:** a State Key Laboratory for Mesoscopic Physics and School of Physics, Peking University Beijing 100871 P. R. China jinglu@pku.edu.cn; b National Water and Energy Center, United Arab Emirates University Al Ain 15551 United Arab Emirates rajwali@uaeu.ac.ae; c School of Microelectronics, Southern University of Science and Technology Shenzhen China salah@sustech.edu.cn; d Department of Physics, University of Poonch Rawalakot Rawalakot 12350 Pakistan; e Collaborative Innovation Center of Quantum Matter Beijing 100871 P. R. China; f Beijing Key Laboratory for Magnetoelectrical Materials and Devices (BKL-MEMD), Peking University Beijing 100871 P. R. China; g Peking University Yangtze Delta Institute of Optoelectronics Nantong 226010 P. R. China; h Key Laboratory for the Physics and Chemistry of Nanodevices, Peking University Beijing 100871 P. R. China; i Beijing Key Laboratory of Quantum Devices, Peking University Beijing 100871 P. R. China

## Abstract

Recently, field-effect transistors (FETs) based on triple-layer InSe have been experimentally fabricated with a channel length of 10–20 nm. They show better performance than Si FETs in terms of transconductance and room-temperature ballistic ratio. Their device performance limits at shorter physical lengths remain to explore. We used the *ab initio* quantum transport simulation method to study monolayer (ML) and bilayer (BL) n-type β-InSe FETs with gate lengths (*L*_g_) of 2 and 3 nm. The on-state current (*I*_on_) values of the ML and BL n-type β-InSe FETs at both 2 and 3 nm *L*_g_ can achieve the International Roadmap Technology for Semiconductors (ITRS) high-performance (HP) device standards. Specifically, the devices achieve *I*_on_ values of 1236 and 648 μA μm^−1^ at *L*_g_ = 2 nm for the ML and BL n-type β-InSe FETs, respectively, surpassing the standard on-state current (528 μA μm^−1^) defined in the 2013 ITRS edition for HP applications. The power-delay product (power consumption), delay time, and energy-delay product (energy consumption) of ML and BL n-type β-InSe also meet the ITRS requirements for HP applications. The ML and BL n-type β-InSe FETs can be potential candidates for future electronics at sub-3 nm physical nodes.

## Abstract 2

Recently, field-effect transistors (FETs) based on triple-layer InSe have been experimentally fabricated with a channel length of 10–20 nm.

## Graphical abstract

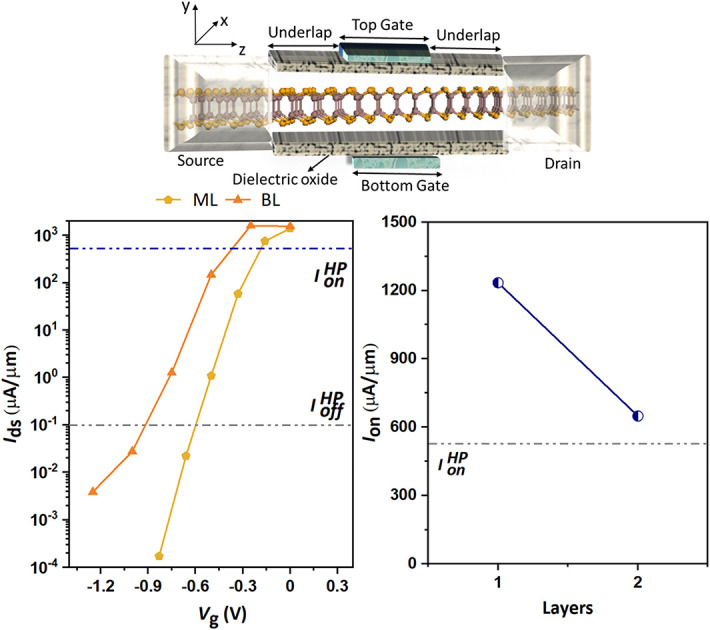

## 1 Introduction

Current silicon-based traditional field-effect transistors (FETs) have approached a critical point due to short-channel effects (SCEs).^1^ SCEs appear drastically in logic switches with ultrashort channel lengths (*L*_ch_) because of poor gate electrostatic control.^2^ As the *L*_g_ decreases, the lateral electric field created by the drain and source electrodes begins to lower the potential barrier for carrier injection at the source electrode (a phenomenon known as drain-induced barrier lowering [DIBL]).^3,4^ This results in a large gate voltage swing (subthreshold swing (SS)), which makes it difficult to turn off the transistor and leads to high power consumption and excessive heat dissipation. These SCEs are a bottleneck for the further development of Si-based devices.^5^ So, it is pressing to find alternative channel materials to alleviate SCEs that could promote ultrasmall FETs at the commercial level.^6^ Among two-dimensional (2D) layered semiconductor materials, the III–VI semiconductor indium selenide (InSe)^7^ has garnered great attention due to its atomic-scale thickness, which provides excellent electrostatic gate control, and its dangling-bond-free and smooth surface, which produces high charge carrier mobility.^8,9^ 2D InSe has a suitable direct bandgap of 1.26 eV and a high carrier mobility of 10^3^ cm^2^ V^−1^ s^−1^.^10^ These advantages make InSe superior to other 2D channel materials for next-generation electronic applications.^11,12^

Recently, Jiang *et al.*^13^ fabricated three-layered 2D InSe ultrashort ballistic transistors with *L*_ch_ values of 10 and 20 nm, using yttrium-doped InSe (Y–InSe) as electrodes to achieve an ohmic contact with a negligible Schottky barrier, and an effective HfO_2_ oxide thickness of 2.6 nm, operated at a bias voltage of 0.5 V. The 10-nm InSe FET exhibited effectively suppressed short-channel effects, with an on-state current of over 1 mA μm^−1^, a low SS of 75 mV per decade, a DIBL of 22 mV per V, and a current on/off ratio (*I*_on_/*I*_off_) of >10^7^. This FET has the best on-state current of 1.43 mA μm^−1^ with *L*_g_ = 20 nm at *V*_bias_ = 0.7 V. So, one interesting question arises: what is the device performance limit of few-layered InSe FETs with a gate-length below 5 nm if an ohmic contact and an ultrathin effective oxide thickness of high-*k* dielectric are achieved?

The relationship between device performance and the number of layers is a research direction of great concern in 2D FETs. Device performance depends on the number of layers: on one hand, the increase in the number of layers of channel material provides additional conduction channels, leading to a high on-state current; on the other hand, an increase in the number of layers results in weak electrostatic gate controllability, thereby degrading the performance of the device. For example, recently, quantum transport simulation of sub-1 nm ML and BL WSe_2_ FETs by Yang in 2025 reported that the device performance of the BL WSe_2_ FET decreases in terms of on-state current (435 μA μm^−1^) compared to the ML WSe_2_ FET (712 μA μm^−1^).^14^ The performance degraded due to the degradation of gate controllability and changes in the band structure. Experimentally, few-layer MoS_2_ FETs with sub-10 nm gate lengths have exhibited good device performance against SCEs with an on/off ratio of 10^5^–10^7^, excellent switching with near-ideal subthreshold swing (SS) of 67–140 mV dec^−1^, and leakage currents of lower than 10^−6^ μA μm^−1^.^15–18^ Significantly, Qi Zhang *et al.* fabricated cutting-edge 1T′-2*H* hetero-phase BL MoTe_2_ field-effect transistors featuring a gate length of 4 nm, which exhibit remarkable switching performance characterized by a subthreshold swing of approximately 73 mV per decade and an on/off current ratio of >10^5^.^19^

Our research plans to explore the theoretical performance limits of monolayer and bilayer β-phase InSe transistors. Herein, we investigated 5-nm-*L*_ch_ double-gated (DG), layer-dependent n-type β-InSe metal–oxide–semiconductor FETs (MOSFETs) by minimizing the *L*_g_ to 2 and 3 nm *via ab initio* quantum transport simulations. Excellent gate controllability was attained in the n-type ML β-InSe FETs. Notably, the on-state currents of 1236 and 1291 μA μm^−1^ at *L*_g_ = 2 and 3 nm, respectively, meet the HP device requirement of the 2028 ITRS standard, as outlined in the 2013 edition.^20^ The best device performance results, such as *I*_on_, delay time (*τ*), and power-delay product (PDP), were obtained when *L*_g_ was scaled down to 2 nm for the optimized layer-dependent n-type β-InSe FETs at a fixed bias voltage of *V*_dd_ = 0.57 V. The on-state currents of the n-type ML and BL β-InSe FETs were 1236 and 648 μA μm^−1^, respectively, at *L*_g_ = 2 nm, both surpassing the required HP ITRS criteria (528 μA μm^−1^). Therefore, our theoretical prediction for InSe in the β-phase confirms it as an excellent channel material candidate for sub-3 nm physical node transistors in high-performance applications.^21,22^

## 2 Computational details

The structural optimization of few-layer β-InSe was conducted using density functional theory (DFT) as implemented in the CASTEP code.^23,24^ The exchange-correlation functional was based on the generalized gradient approximation (GGA) in the form of the Perdew–Burke–Ernzerhof (PBE).^25^ The plane-wave basis set with an energy cut off of 450 eV was selected at a temperature of 300 K. The structure was relaxed using norm-conserving pseudopotentials, with a stress tolerance of 0.001 eV Å^−1^ and a force tolerance of 0.01 GPa between two points. The *k*-point mesh for structure relaxation was set to 11 × 11 × 1. The Heyd–Scuseria–Ernzerhof (HSE) exchange–correlation functional was adopted for band structure calculations.^26^ The spin–orbit interaction was excluded. A dense Monkhorst–Pack *k*-point mesh of 31 × 31 × 1 was used for band structure calculations. To eliminate artificial interactions in supercells due to periodic boundary conditions and to account for van der Waals (vdW) interactions, two corrections are considered: the DFT-D2 correction and the dipole correction.^27,28^ A 15 Å vacuum buffer space was considered along the *z*-direction to weaken interactions between the adjacent slabs in the 2D layered structure. After optimizing the primitive cell, a rectangular supercell was formed according to the transport orientation.

The transport properties of the FET device configurations were simulated with the density functional theory (DFT) combined with the nonequilibrium Green's function (NEGF), as employed in QuantumATK 2022.^29^ The exchange–correlation potential in the form of GGA-PBE was applied in all the device transport calculations. DFT-GGA is good for the single-electron approximation and tends to underestimate the band gap of intrinsic semiconductors. However, the channel is surrounded by a gate and dielectric, which strongly screens the electron–electron interaction.^30^ The double-zeta (*ζ*) polarized (DZP) basis set was employed to accurately capture the shape of the molecular orbitals. The temperature was fixed at 300 K, and the real-space density mesh cut-off energy was set to 80 hartree. The DFT-GGA implementation was rigorous enough to determine the carrier transport in the FET configuration. The periodic, Neumann, and Dirichlet-type boundary conditions were used on the boundaries along the transverse, vertical, and transport directions, respectively.^31^ The β-InSe channel is located in the *xz*-plane, while transport is along the *z*-direction (Fig. 2(a)). To guarantee that the electrostatic potential in the central region is adequately screened, we prolonged the electrodes at the interface by extending their unit cell two times (11.5 Å) in the *z*-direction.^32^ The periodicity of the channel in the *x*-direction (the plane of the β-InSe sheet, perpendicular to the transport direction *z*) was set to 32 *k*_*x*_ points, and *y* was the confinement direction. We choose a dense *k*-point mesh along the periodic direction because electronic states with periodic boundaries are characterized precisely by selecting a fine sampling number of the *k*-points.^33^ The injection of electrons/holes was set as a 32 × 1 × 175 Monkhorst–Pack *k*-point mesh in the *x*, *y*, and *z* directions, respectively, within the irreducible Brillouin zone.

In the two-probe MOSFET model, the device consists of a central region (including the scattering region) and the electrode region; the left and right electrodes are semi-infinite. The impact of the electrodes on the surface of the scattering region is taken into account in the form of self-energies ∑_1/r,*k*‖_,^34^ which are calculated from the electrode Hamiltonians and coupling Hamiltonians. The reciprocal lattice vector *k*_‖_ points along the surface-parallel direction (orthogonal to the transmission direction) in the irreducible Brillouin zone (IBZ).^35^ The Hamiltonian matrix *H* of the central region, together with the overlap matrix *S*, generates the retarded Green's function matrix that includes the self-energies from the electrodes:

1 *G*_*k*_‖__(*E*) = [(*E* + *iδ*_+_)*S*_*k*_‖__ − *H*_*k*_‖__ − ∑_l,*k*_‖__ − ∑_r,*k*_‖__]^−1^,

where *δ*_+_is an infinitesimal positive number. The transmission coefficient *T*_*k*_‖__(*E*) is the average of the *k*-dependent transmission coefficient over the IBZ, defined as follows:2
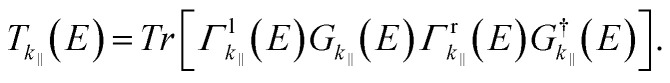
Here, the gamma function 
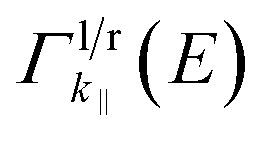
 stands for the energy-level broadening 
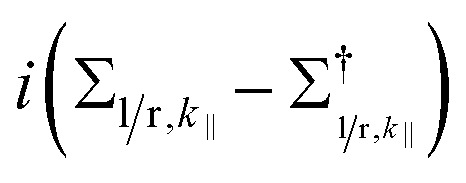
 of the right and left electrodes, expressed as self-energies, and the retarded *G*_*k*_‖__(*E*) and advanced 
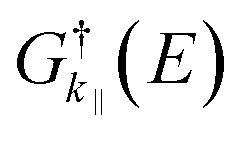
 Green's functions are obtained from the NEGF method. Using the Landauer–Büttiker formula,^36^ the drain current *I*_ds_ at a given gate voltage *V*_g_ and bias voltage *V*_b_ is calculated as follows:3
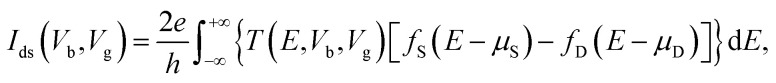
where *f*_S/D_ is the Fermi–Dirac distribution function, and *μ*_S/D_ is the electrochemical potential of the source and drain electrodes.

The DFT method based on the single-electron approximation is used to model electron behavior in the FET configuration. The reliability of the *ab initio* quantum transport simulation is validated by comparing the calculated band gap of ML MoSe_2_, which is 1.52 eV at the DFT-GGA level, with an experimentally obtained value of 1.58 eV from angle-resolved photoemission spectroscopy.^37^ It is also verified by a predicted high on-state current of 1.5 mA μm^−1^ for the ML InSe FET with *L*_g_ = 7 nm, which well matches the observed value of 1.5 mA μm^−1^ for the trilayer β-InSe FET with *L*_g_ = 20 nm.^38,39^

## 3 Results

**Fig. 1:**
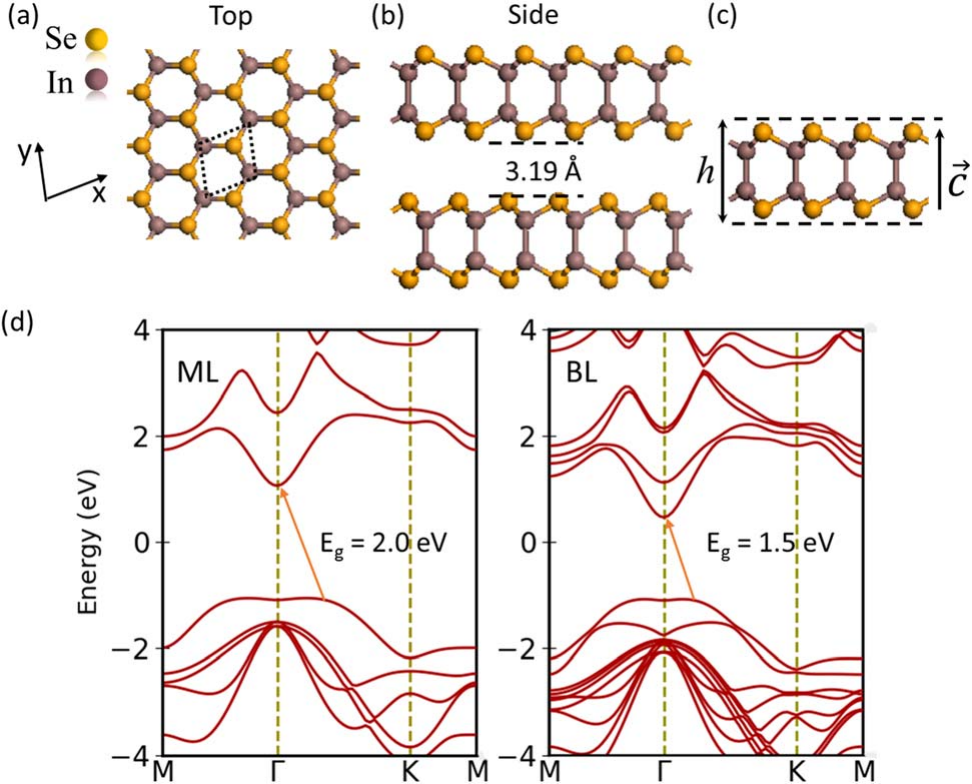
(a) Top and side views of the bulk 2H-phase β-InSe structure. (b) The Layer stacking and layer distance. (c) The thickness of the ML InSe and the lattice vector along the *c*-direction. (d) The electronic band structures of ML and BL β-InSe at high-symmetric points in the irreducible Brillouin zone. The positions of the valence band maxima, conduction band minima, and the band gap (*E*_g_) are indicated.

The ML β-phase InSe has a honeycomb lattice composed of a quadruple atomic sheet arranged in the order Se–In–In–Se. The structure exhibits strong covalent bonding within each layer, while weak van der Waals (vdW) forces exist between adjacent layers.^40,41^ The hexagonal primitive cell is labeled with a black-colored frame, as shown in Fig. 1(a), and shows the AB stacking sequence^40^ in Fig. 1(b). The optimized lattice parameters of ML β-InSe are as follows: *a* = 4.08 and *c* = 25.85 Å, which are consistent with previous experimental^5^ and theoretical results.^42,43^Fig. 1(c) exhibits the Se–Se height (*h*) of the ML β-InSe and the layer distance of 5.31 (ref. 42) and 3.19 Å,^2^ respectively. The layered β-InSe structure shows a decrease in bandgap due to the quantum confinement effect. With the increase in the number of layers, the valence and conduction bands split into sub-bands, resulting in a reduced bandgap. The results illustrated in Fig. 1(d) indicate that the bandgap of β-InSe decreases from monolayer to bulk.^5^

In the β-InSe band structures, the conduction band minimum (CBM) is at the *Γ*-point, while the valence band maximum (VBM) is at the *Γ*–*K* direction, resulting in an indirect bandgap. It is noteworthy that the electronic band structure of β-InSe has a conversion from direct to indirect bandgap with a decrease in the number of layers.^44^ The band structures of the ML and BL hexagonal β-InSe, calculated with DFT-HSE approaches, are 2.0 and 1.5 eV, respectively, which is consistent with a previous report.^26^ The band near the conduction band maxima is steeper than the valence band minima. It identifies a lighter electron-effective mass than that of the hole, which is clear evidence of high electron carrier current in the n-type β-InSe MOSFETs. In Fig. 1(d), from the band dispersion spectrum point of view, the electron effective mass 
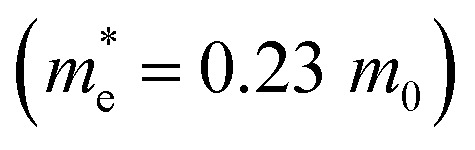
 is smaller than the hole effective mass 
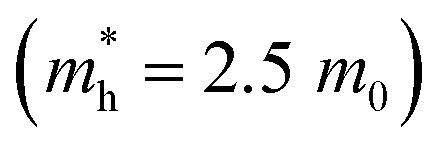
. The effective mass is the reciprocal of the curvature of the band dispersion spectrum. A small effective mass increases carrier velocity 
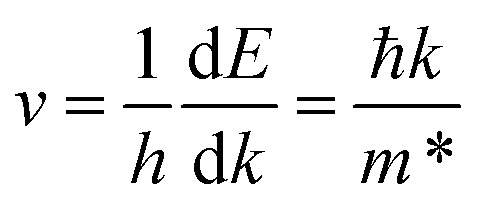
, and thus the current.

**Fig. 2:**
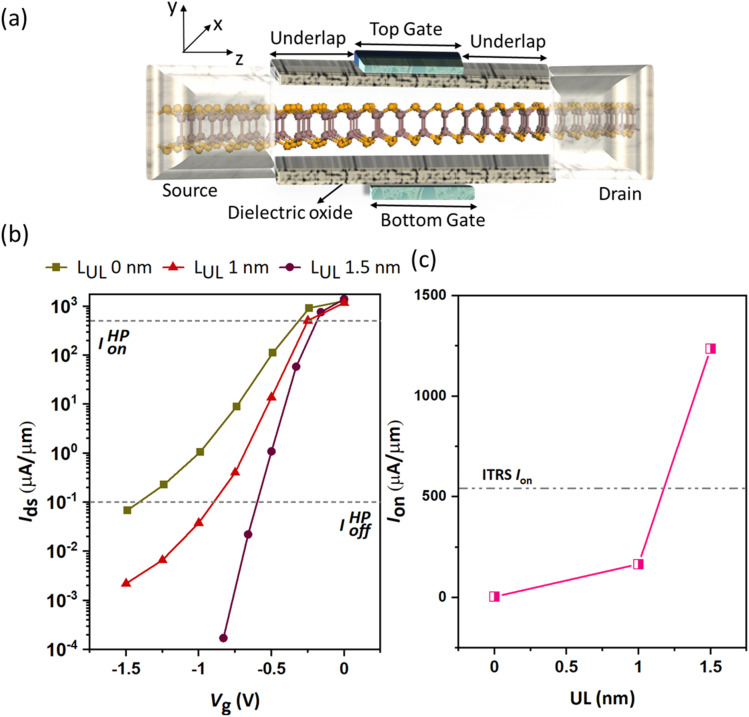
(a) Schematic of the double gate (DG) InSe MOSFET with the underlap length on both sides of the gate as an extension region of the channel. The source and drain are symmetrically n-type doped. (b) Transfer characteristic and (c) *I*_on_ at different underlap lengths of *L*_UL_ = 0, 1, and 1.5 nm for the n-type ML DG β-InSe MOSFETs.

A schematic view of our double-gated (DG) β-InSe device with a 5 nm channel is illustrated in Fig. 2(a). In a FET, the potential generated by the source and drain consistently competes with the potential induced by the gate. The lesser the influence of the source and drain on the gate, the more effectively it can be controlled and adjusted by the gate.^45,46^ The length to which the electrical potential from the source and drain penetrates the channel is defined as the natural length *λ*:4
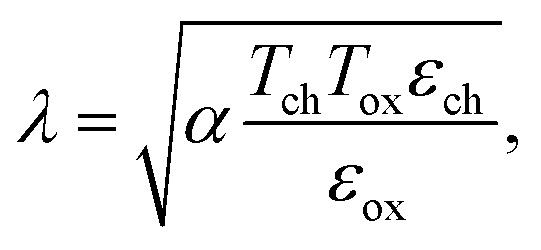
where *α* is the gate coefficient, while *ε*_ch_/*ε*_ox_ and *T*_ch_/*T*_ox_ is the dielectric constant and thickness of the gate oxide and channel, respectively. The *λ* is calculated to be 0.41 and 0.67 nm for ML and BL β-InSe, respectively, using *ε*_ch_ = 8.15. The increase in *λ* specifies a reduction in gate controllability over the channel due to the increasing number of layers. The drain and source electrodes are heavily doped with an n-type doping concentration (*N*_S/D_) of 1 × 10^13^ cm^−2^. A high dielectric material (high-*k*), HfO_2_, with a dielectric constant of 20 and an effective oxide thickness (EOT) of 1.5 and 1.7 nm for *L*_g_ = 2 and 3 nm, respectively, is employed. To further enhance the performance of devices, the device structure with underlap length (*L*_UL_), *i.e.*, an ungated section, is considered in the n-type few-layer β-InSe MOSFETs. The optimal *L*_UL_ of 1.5 and 1 nm is symmetrically selected on both sides of the metal gate for *L*_g_ = 2 and 3 nm, respectively. However, the whole length of the channel is equivalent to the sum of the underlap lengths and gate length, formulated as (*L*_ch_ = *L*_g_ + 2*L*_UL_), and does not exceed 5-nm. The supply voltages (*V*_dd_ = *V*_bias_) are fixed to 0.57 and 0.59 V, according to the HP ITRS 2013 standard for *L*_g_ = 2 and 3 nm, respectively. In transfer curves, the off-state voltage (*V*_off_) is the gate voltage at which the off-state current (*I*_off_) is just 0.1 μA μm^−1^ for the HP-ITRS devices (2013 version) for the target year 2028. However, *I*_on_ can be evaluated at the specific on-state gate voltage (*V*_g(on)_ = *V*_g(off)_ + *V*_dd_) for n-type devices, where *V*_g_ is the gate voltage.

### 3.1 Current

**Fig. 3:**
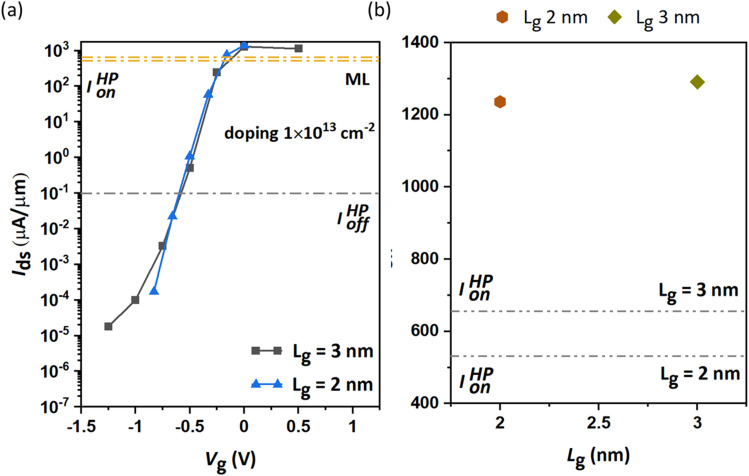
(a) Transfer characteristics and (b) *I*_on_ at different gate lengths of *L*_g_ = 2 and 3 nm for the n-type ML β-InSe MOSFETs.

The conventional FET works on the operational principle of controlling the drain current (*I*_ds_) by changing the gate voltage (*V*_gs_) between the gate and the source. High switching speed demands a quick response from the FET to change in *V*_g_. This requires strong gate controllability in the FET devices. In this study, we begin our investigation of n-type doping (1 × 10^13^ cm^−2^) in ML β-InSe with *L*_g_ = 2 nm to get the transfer characteristics under *L*_UL_ = 0, 1, and 1.5 nm, as shown in Fig. 2(b). It can be observed that the on-state current increases sharply with an increase in the UL region, reaching 4, 166, and 1236 μA μm^−1^ for the n-type ML β-InSe, as illustrated in Fig. 2(c). We selected the optimal UL length of 1.5 nm for different *L*_g_ values of 2 and 3 nm. The current (*I*_ds_) transfer characteristics for the n-type ML β-InSe MOSFET devices at *L*_g_ = 2 and 3 nm are shown in Fig. 3(a). The on-state currents for the gate lengths of 2 and 3 nm are nearly comparable, at 1236 and 1291 μA μm^−1^, respectively, and outperforms the HP ITRS device requirement of *I*_on_ (528 and 650 μA μm^−1^), as shown in Fig. 3(b). The evidential best-performing transfer characteristics (*I*_on_) at different gate lengths encourage us to choose InSe MOSFETs with *L*_g_ = 2 nm for layer-dependent device performance.

**Fig. 4:**
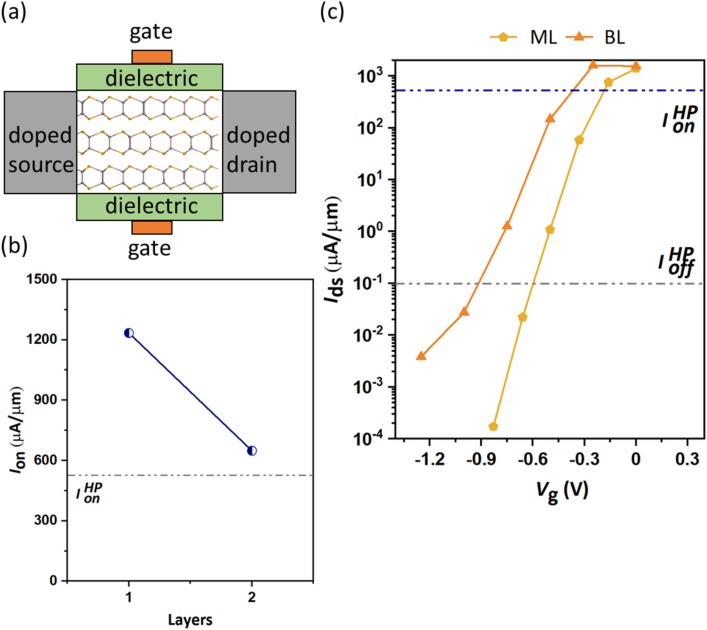
(a) Schematic of the layered InSe channel MOSFET. (b) Transfer characteristics at different layers with a fixed *L*_g_ of 2 nm. (c) Figure of merit, *I*_on_ for the n-type devices.

The schematic view of the layered DG β-InSe channel configuration is shown in Fig. 4(a). The crucial figure of merit for logic devices, *I*_on_, is obtained for ML and BL n-type β-InSe MOSFETs, as presented in the layer-dependent transfer characteristics in Fig. 4(b). All the calculated transfer curves for the n-type β-InSe MOSFETs could easily reach the HP off-state current (0.1 μA μm^−1^) for devices with *L*_g_ = 2 nm, and the on-state current values of the ML and BL β-InSe devices surpass the HP ITRS device requirements (528 μA μm^−1^). Specifically, *I*_on_ for ML and BL n-type β-InSe FETs are 1236 and 648 μA μm^−1^, respectively, compared to the HP ITRS target for 2028. The gradual decrease in on-state currents of the layer-dependent n-type β-InSe FETs with increasing layer number is illustrated in Fig. 4(c). The relationship between the number of layers often leads to more conductive channels as the number of layers increases. On the other hand, an increase in channel thickness may lead to a reduction in the gate control ability. Based on the figure of merit, *I*_on_, the performance of layer-dependent n-type β-InSe MOSFETs is predicted to degrade with an increasing number of layers, which may be attributed to the weakened gate control ability associated with the increase in the number of layers of the channel material.

**Fig. 5:**
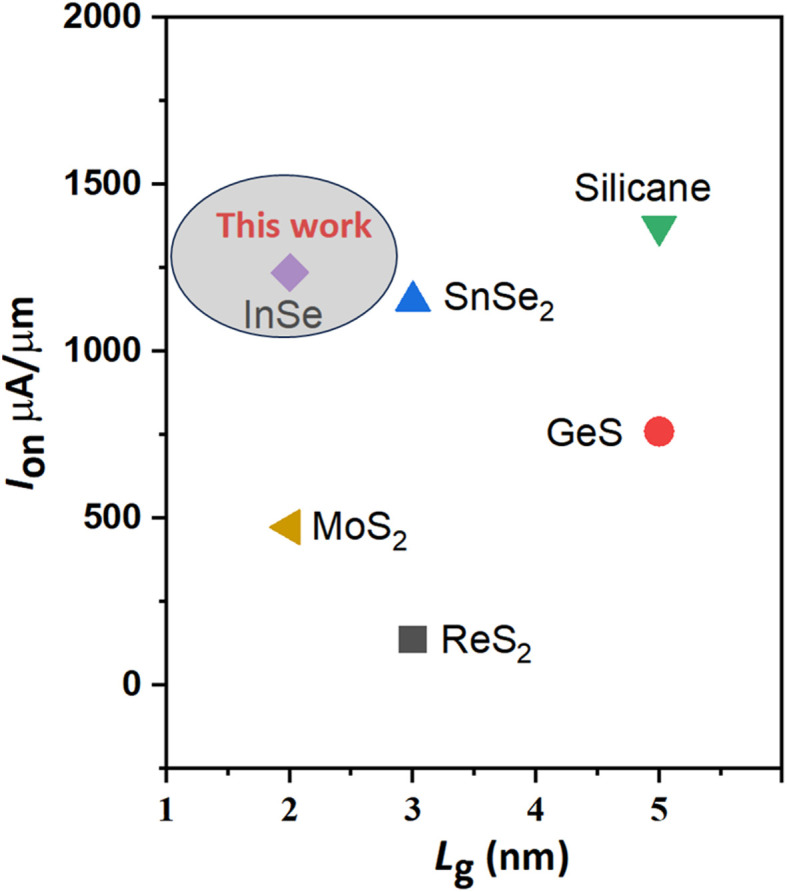
*I*_on_ *versus L*_g_ of the ML DG MOSFETs at sub-5 nm *L*_g_ and different UL structures for HP devices.

Notably, the performance decline when moving from ML to BL β-InSe can be explained by several fundamental physical mechanisms. These mechanisms pertain to alterations in the electronic structure, carrier mobility, and interlayer interactions that take place during the transition from ML to a BL material. ML β-InSe features a direct bandgap. However, upon transitioning to BL β-InSe, the system frequently shifts to an indirect bandgap. This transition renders ML β-InSe more advantageous for certain applications in comparison to BL β-InSe. In BL β-InSe, the electronic structure is influenced by interlayer interactions, leading to energy level splitting and a transition to an indirect bandgap. Typically, carrier mobility diminishes as the number of layers increases in 2D materials. This reduction is primarily due to interlayer coupling that occurs in BL configurations. Specifically, in the BL, carriers encounter additional scattering from interlayer interactions, which are not present in the ML scenario. Consequently, the electrical conductivity and carrier mobility in BL β-InSe are generally lower than those in ML β-InSe, thereby constraining its performance in transistor applications or high-speed electronics. Our calculated *I*_on_ for n-type ML and BL β-InSe FETs with *L*_g_ = 2 nm is comparable to that of other ML 2D-material MOSFETs with longer *L*_g_, for example, MoS_2_,^47^ ReS_2_,^48^ GeS,^49^ SnSe_2_,^50^ InSe,^12,51^ and silicane,^52^ as shown in Fig. 5.

**Fig. 6:**
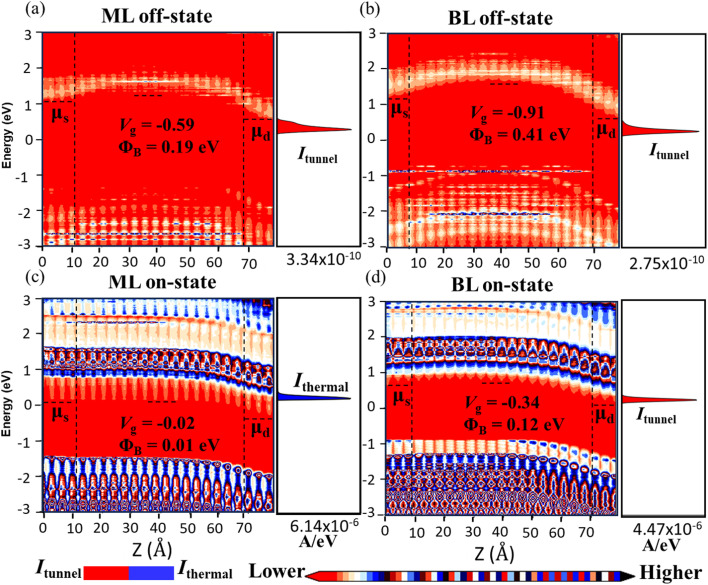
Local device density of states and spectral currents of the ML and BL n-type β-InSe MOSFETs with *L*_g_ = 2 nm at the (a and b) off- and (c and d) on-states, with *L*_UL_ = 1.5 nm. *V*_dd_ = *μ*_d_ − *μ*_s_ = 0.57 eV.

The gate modulation and current variation mechanisms are unveiled by the position-fixed local density of states (LDOS) and the current spectrum of 2-nm-*L*_g_ n-MOSFETs for ML and BL β-InSe at a bias of *V*_dd_ = 0.57 V, as shown in Fig. 6. We investigated the layered structure of β-InSe by simulating device performance at different gate voltages to analyze the on- and off-state currents. The high value of *Φ*_B_ leads to a sharp decrease in current with increasing gate voltage. The energy difference between the Fermi level of the source and the conduction band minima (CBM) of the channel is referred to as the electron activation energy (*Φ*_B_). Under a gate modulation of 0.57 V, *Φ*_B_ decreases gradually from off-state values of 0.19 and 0.41 eV to on-state values of 0.01 and 0.12 eV, respectively, corresponding to gate voltages ranging from off-state −0.59 and −0.91 V to on-state −0.02 and −0.34 eV, in increasing order from the ML to BL n-type β-InSe, respectively. The CBM within the channel region increases with an increasing number of layers in the β-InSe channel material. This leads to a reduction in off-state current from ML to BL, recorded as 3.34 × 10^−10^ and 2.75 × 10^−10^ A eV^−1^, respectively (0.1 μA μm^−1^ for the ITRS HP goal). Conversely, the on-state currents decrease from ML to BL n-type β-InSe, recorded as 6.14 × 10^−6^ and 4.47 × 10^−6^ A eV^−1^, respectively. Usually, *I*_on_ is composed of both thermionic current and tunnelling current; however, in our investigation of layer-dependent n-type β-InSe, the transport characteristics are primarily influenced by tunneling current (*I*_tunnel_), except for the on-state n-type ML β-InSe device. The lack of thermal current indicates a significant barrier height in the source-to-drain region, resulting in minimal contribution from thermal current to the overall current. This evidence is verified by the current spectra shown in Fig. 6. For the on-state LDOS of the ML β-InSe MOSFET, the barrier height is reduced to zero. Therefore, the current saturates, and the thermal current *I*_threm_ becomes dominant.

**Fig. 7:**
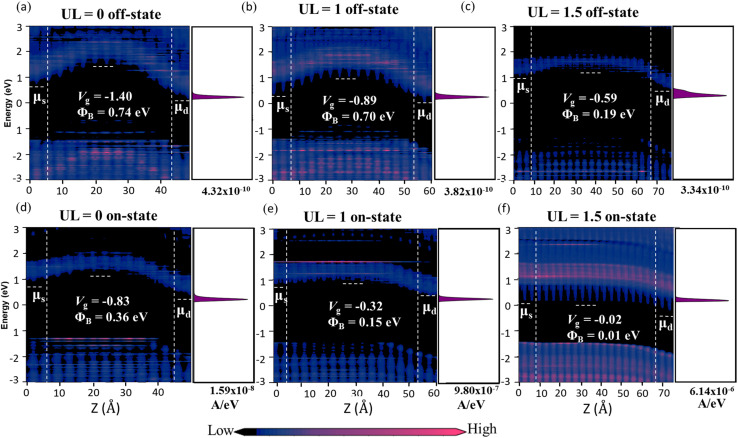
Local device density of states and spectral currents of the ML n-type β-InSe MOSFETs at different UL structures with *L*_g_ = 2 nm at the (a–c) off- and (d–f) on-states. *V*_dd_ = *μ*_d_ − *μ*_s_ = 0.57 eV.

The local density of states and spectral current are illustrated in Fig. 7 to assess the performance of transistors across various UL structures. As *L*_UL_ increases from 0 to 1 and 1.5 nm, the effective channel length of the device increases, tunneling leakage current reduces (Fig. 7(a–c)), and the on-state current increases (Fig. 7(d–f)). The improvement in gate control due to longer *L*_UL_ is reflected in the enhanced modulation of the band edge locations at smaller *V*_g_ values. Therefore, the short-channel effects are suppressed significantly. In this investigation, we take n-type ML β-InSe DG MOSFETs with *L*_g_ = 2 nm and varying UL lengths as an example. By keeping the off-state current fixed at 0.1 μA μm^−1^, the energy barrier is high at 0.74, 0.70, and 0.19 eV for *L*_UL_ = 0, 1, and 1.5 nm, respectively, and the three spectral currents are of the same order of magnitude. The CBMs of the n-type ML β-InSe MOSFETs move downward in the channel region under gate modulation of 0.57 eV, and hence the devices turn into the on-state. The *Φ*_B_ for *L*_UL_ = 0 nm is 0.36 eV, which decreases significantly to 0.15 and 0.01 eV for *L*_UL_ = 1 and 1.5 nm, respectively. It can be predicted that enhanced electrostatics induced by increasing UL length favor a high on-state current. The magnitude of the spectral current indicates the increase in current with long *L*_UL_: 1.59 × 10^−8^, 9.80 × 10^−7^, and 6.14 × 10^−6^ for *L*_UL_ = 0, 1, and 1.5 nm, respectively. The current mainly comes from transmissions above the source chemical potential (*μ*_s_) in terms of spectral current. The on-state current enhances rapidly to 4, 166, and 1236 μA μm^−1^ for *L*_UL_ = 0, 1, and 1.5 nm, respectively.

### 3.2 Subthreshold swing

**Fig. 8:**
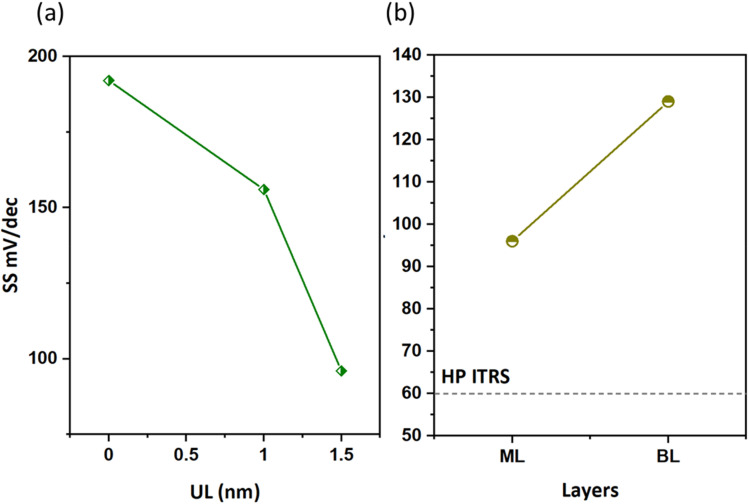
Subthreshold swing for n-type DG β-InSe MOSFETs with different UL structures (a) and for the layers of the n-type DG β-InSe MOSFETs (b).

Subthreshold swing (SS) is an important index for assessing gate controllability in MOSFETs within the subthreshold region. It can be described as the change in gate voltage necessary to change the drain current by one order of magnitude. It is formulated as 
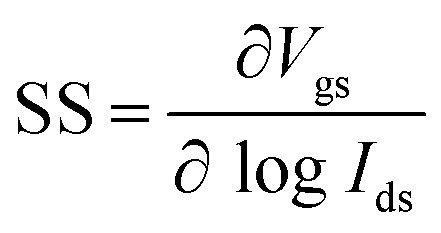
. The lowest limit of SS is 60 mV dec^−1^ according to the “Boltzmann tyranny”.^45^ We extracted SS from the transfer characteristics of ML and few-layered n-type DG β-InSe MOSFETs with different UL values, as shown in Fig. 8(a and b). SS decreases rapidly as *L*_UL_ increases from 0 to 1 and 1.5 nm. SS at UL = 0 and 1 nm is calculated as 192 and 156 mV dec^−1^, respectively, while SS decreases rapidly to 96 mV dec^−1^ when *L*_UL_ is further increased to 1.5 nm at the same gate length, as shown in Fig. 8(a). By implementing a longer UL length, SS experiences a significant reduction. The reason for adopting an elongated UL structure lies in its ability to enhance the effective channel length, thereby mitigating leakage through the source-to-drain electrode and improving the efficiency of gate electrostatics. Notably, to achieve a small value of SS, we suggest a long UL structure, particularly for *L*_g_ 2 nm in the fabrication of layered β-InSe MOSFETs. For n-type DG β-InSe MOSFETs, SS is 96 for ML β-InSe FET devices. As the number of layers increases to BL, SS increases rapidly to 129 mV dec^−1^ due to short-channel effects, as shown in Fig. 8(b). Large variations in gate voltage are required to switch the transistor between the off- and on-states. It also predicts that the source-to-drain leakage current is more effectively suppressed in ML than in BL β-InSe MOSFETs. Increasing the number of layers inhibits tunnelling between the source and the drain, as it leads to an increase in channel thickness and reduction in electrostatic control. Thereby, a smaller SS presents better gate controllability of the channel. SS is expressed as5
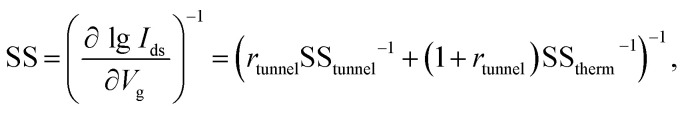
where 
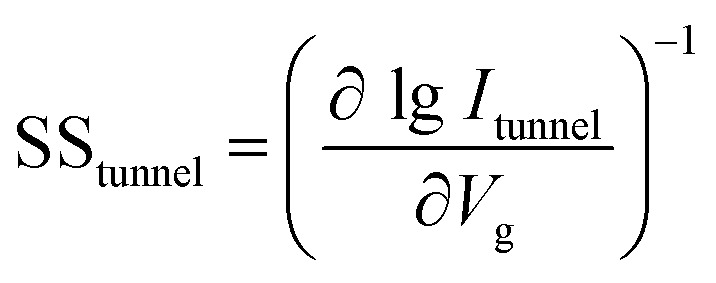
, 
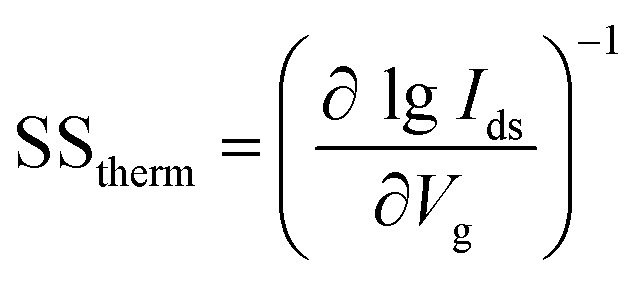
 and 
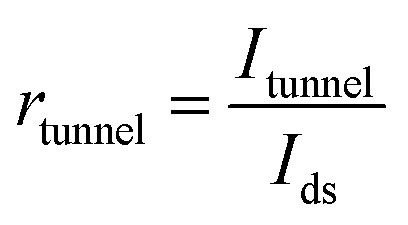
. In ultrasmall-channel MOSFETs, tunnelling current is a major contributor. So, *r*_tunnel_ ≠ 0, and SS is less likely to approach the thermal limit of 60 mV dec^−1^. In case of long channel lengths, the current comes from thermionic injection, so *r*_tunnel_ = 0, and SS reaches the lower thermionic limit (60 mV dec^−1^).

**Fig. 9:**
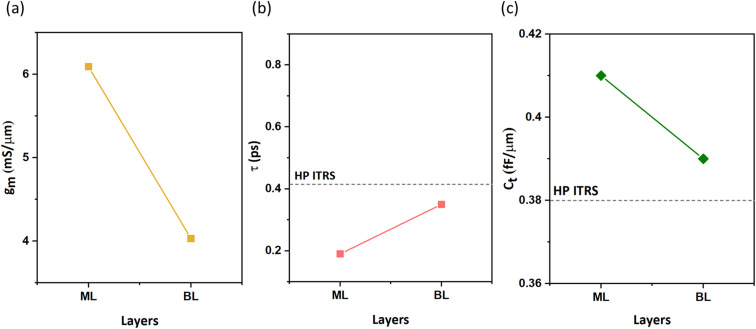
(a) Transconductance, (b) total capacitance (c) and intrinsic delay time of the ML and BL n-type DG β-InSe MOSFETs at *L*_g_ = 2 nm.

We evaluate device performance using another critical parameter: transconductance (*g*_m_). In the subthreshold region, *g*_m_ is to estimate gate control for different layers of n-type β-InSe FETs. It is defined as the change in current per unit change in gate voltage, which can be formulated as 
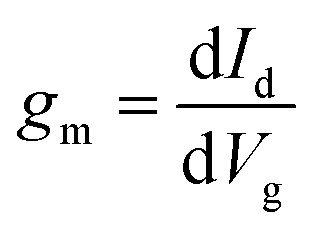
. The *g*_m_ values for n-type ML and BL DG β-InSe MOSFETs are plotted in Fig. 9(a). For the n-type ML and BL β-InSe FET devices, *g*_m_ values are 6.09 and 4.03 mS μm^−1^, respectively. In layered β-InSe, *g*_m_ gradually decreases as the number of layers increases. A large value of *g*_m_ indicates excellent gate control and explains the large *I*_on_ observed for the n-type ML and BL β-InSe FETs, which is larger than the HP ITRS on-state current standard. The gradual decrease in *g*_m_ from ML to BL β-InSe FETs reflects weak gate control in the channel.

### 3.3 Intrinsic delay time and power consumption

**Fig. 10:**
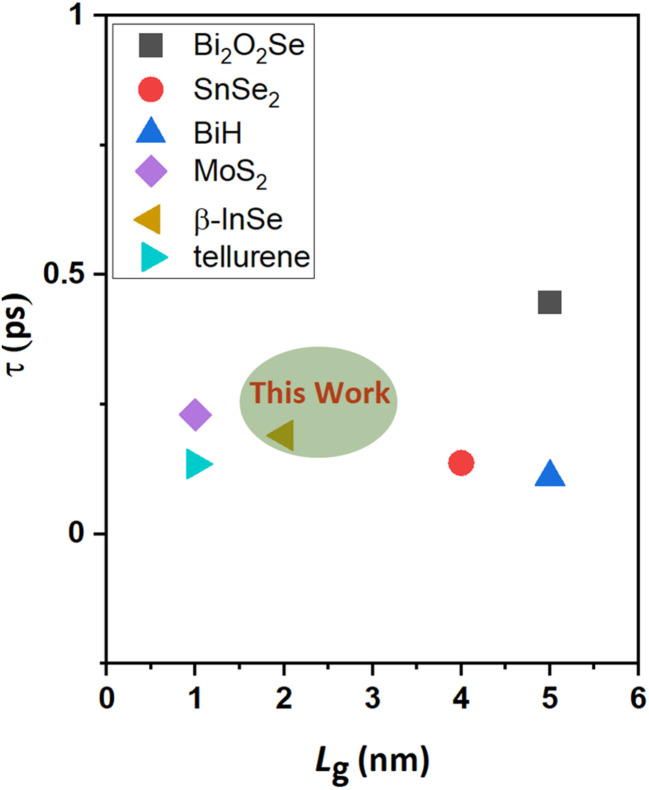
Comparison of the switching speed of the ML n-type DG β-InSe MOSFETs with other 2D-material FETs at sub-5 nm *L*_g_.

To measure the performance limits of few-layer n-type β-InSe FETs, the other figures of merit, such as delay time, total capacitance *C*_t_, and power dissipation (PDP), are listed in Table 1. These figures of merit are shown in Fig. 9(b). The intrinsic 
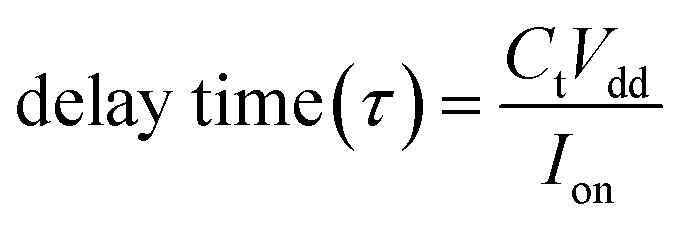
 is a valid metric to evaluate device switching speed. The total capacitance *C*_t_ is the sum of the gate capacitance (*C*_g_) and the fringing capacitance (*C*_f_ = 2*C*_t_). So, the total capacitance is three times the gate capacitance, 
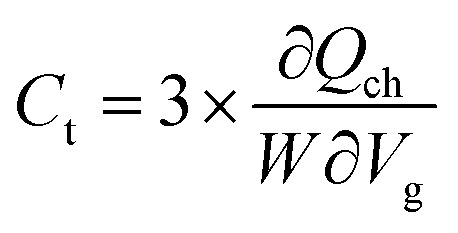
, where ∂*Q*_ch_ = *Q*_on_ − *Q*_off_ is the total charge in the central region of the device, where ∂*V*_ch_ = *V*_on_ − *V*_off_, and *W* is the width of the 2D β-InSe sheet. The *C*_t_ of n-type ML and BL β-InSe FETs are calculated as 0.41 and 0.39 fF μm^−1^, respectively. *C*_t_ values for the n-type ML and BL β-InSe FETs can satisfy the ITRS requirement of 0.38 fF μm^−1^ for HP devices, as outlined in the 2013 version standard. The *C*_t_ values of n-type β-InSe FETs decrease with increasing number of layers, as shown in Fig. 9(c). Additionally, *τ* is proportional to *C*_t_ and inversely proportional to *I*_on_. The ML and BL n-type β-InSe FETs with *L*_g_ = 2 nm show *τ* values of 0.190 and 0.350 ps, corresponding to currents of 1236 and 648 μA μm^−1^, respectively, and can meet the set standard for the HP ITRS (0.410 ps) devices. The small values of *τ* indicate superior performance in terms of switching capability. However, large values of the delay time result in low switching speeds for the transistor applied in a digital circuit. Our calculated *τ* for ML n-type β-InSe FETs shows a switching rate comparable to sub-5 nm *L*_g_ ML 2D-material FETs with long channel lengths, as illustrated in Fig. 10.

**Table 1:** Ballistic performance of n-type β-InSe DGFETs against the ITRS 2013 requirements for HP transistors of the next decades. *L*_g_: gate length. UL: underlap length. *I*_on_: on-state current. SS: subthreshold swing. *g*_m_: transconductance. *C*_t_: total capacitance. *τ*: delay time. PDP: power-delay product. EDP: energy-delay product

Parameters	*L*_g_ (nm)	UL (nm)	Doping (cm^−2^)	*I*_off_ μA μm^−1^	*I*_on_ μA μm^−1^	SS mV dec^−1^	*C*_t_ fF μm^−1^	*g*_m_ mS μm^−1^	*τ* ps	PDP fJ μm^−1^	EDP Js μm^−1^
ITRS	2			0.1	650/528		0.38		0.410	0.12	0.49 × 10^−28^
ML n-type	2	0	1 × 10^13^	0.1	4	192					
ML n-type	2	1	1 × 10^13^	0.1	166	156					
ML n-type	2	1.5	1 × 10^13^	0.1	1236	96	0.41	6.09	0.190	0.13	2.4 × 10^−29^
BL n-type	2	1.5	1 × 10^13^	0.1	648	129	0.39	4.03	0.350	0.12	4.2 × 10^−29^
ML n-type	3	1.5	1 × 10^13^	0.1	1291	82	0.60	7.26	0.272	0.20	4.9 × 10^−29^

**Fig. 11:**
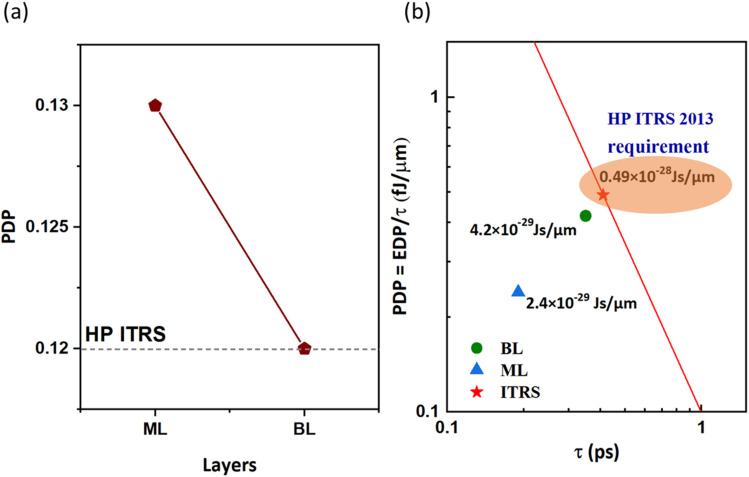
(a) Power-delay product (PDP) of the n-type ML and BL β-InSe FETs at *L*_g_ = 2 nm and UL = 1.5 nm and (b) benchmarks of power dissipation (PDP = EDP/*τ*) *vs.* the effective delay time (*τ*) of the n-type ML and BL β-InSe FETs against the ITRS 2013 edition (represented by red star) for HP applications.

Power dissipation serves as a crucial metric for assessing energy consumption during a single on-off switching event. PDP can be determined using the equation PDP = *V*_dd_*I*_on_*τ* = *C*_t_*V*_dd_^2^. Fig. 11(a) illustrates the relationship between PDP and the number of layers against the ITRS 2013 standard for high-performance applications. According to the ITRS, PDP is proportional to *C*_t_ at a fixed *V*_dd_ = 0.57 V. In Fig. 11(a), PDP decreases from ML to BL n-type β-InSe FETs. Owing to the monotonic decline in *C*_t_, the n-type β-InSe FETs exhibit a symmetry reduction in PDP for the ML and BL n-type β-InSe FET configurations, with calculated values of 0.13 and 0.12 fJ μm^−1^, respectively. The calculated PDP for the ML n-type β-InSe FET is 0.1 points higher than the HP ITRS standard value of 0.12 fJ μm^−1^ for the target year 2028. PDP values for the BL n-type β-InSe FETs align with the standard value of 0.12 fJ μm^−1^. PDPs are close to the HP IRDS standard, which suggests a low power consumption and fast switching compared to the ML MoS_2_ MOSFET (0.195 fJ μm^−1^).^53^ For transistors, fast-switching speed and low power dissipation are preferred. However, these two goals frequently present a conflict, making it difficult to accomplish both at the same time. A high *I*_on_ improves switching speed and power consumption, as reflected in the data shown in Table 1.

By taking switching speed and power dissipation into consideration, the energy-delay product (EDP) can be calculated by the following formula: 
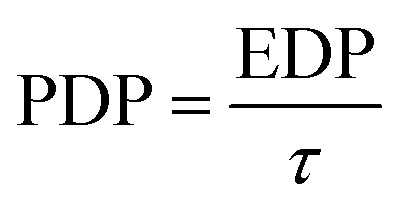
. The smaller the EDP, the better the device performance. EDPs for the layered structure configuration of the n-type β-InSe FETs are shown in Fig. 11(b). In this figure, the ITRS 2013 standard for the 2028 target is represented by a red star. The red line represents the equation 
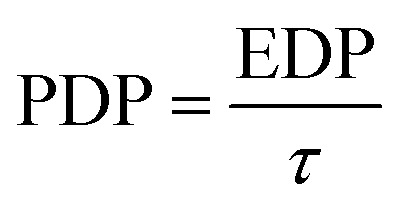
, where the EDP value is the requirement of the HP ITRS (0.492 × 10^−28^ Js μm^−1^) for the target year 2028. The EDPs of ML and BL n-type β-InSe FETs are 2.4 × 10^−29^ and 4.2 × 10^−29^ Js μm^−1^, respectively, fulfilling the HP ITRS standard (0.492 × 10^−28^ Js μm^−2^). EDP falls below the ITRS 2013 requirements, suggesting a promising future for n-type β-InSe FETs. To assess the performance of ML and BL n-type β-InSe MOSFETs, device performance metrics, especially *I*_on_, *τ*, and PDP, are analyzed and compared with those of ML FETs based on other 2D heterostructure materials. All comparative data are derived from theoretical calculations using ballistic transport theory. The *I*_on_ of the ML n-type β-InSe FET (1236 μA μm^−1^) is higher than that of the BL n-type β-InSe FET (648 μA μm^−1^) and relatively higher than a few other 2D-material FETs with long *L*_g_ values, as shown in Fig. 4(b).^5^ Notably, *τ* calculated for ML n-type β-InSe FETs shows a much smaller value, at 0.190 ps, which is lower than the ITRS HP standard, while the switching speed of the M*L* n-type β-InSe FET is comparable to that of other 2D-material FETs with long *L*_g_ values, as shown in Fig. 10. The PDP of ML n-type β-InSe FET is 0.13 fJ μm^−1^, which is high and has a direct relation to the on-state current, while BL n-type β-InSe FETs own a low PDP value of 0.12 fJ μm^−1^ because of their reduced on-state current. PDP of 2D heterostructure is 0.018 fJ μm^−1^ for HP applications.^47–50,54^ The EDP for the ML n-type β-InSe FET (2.4 × 10^−29^ Js μm^−1^) is observed to be lower than the ITRS HP standard, as well as certain few-layer n-type β-InSe FETs. The high value of EDP is observed in the MoS_2_ FET with a channel length of 10 nm, while the best performance is attributed to the black phosphorus (BP) FET.^55^ The EDP values of the few-layer n-type β-InSe FETs are the average of the above devices, demonstrating excellent performance.

## 4 Discussion

In the search for 2D-material FETs that can replace conventional Si FETs, no 2D semiconductor-based experimental FETs have exhibited performance that could exceed that of Si FETs, while few-layer InSe has emerged as an interesting option. In 2D material FETs, achieving both low-resistance ohmic contacts and ultrathin effective oxide thicknesses simultaneously present significant challenges. Recently, Jiang *et al.* in 2023 (ref. 13 and 56) fabricated an ohmic-contact ballistic InSe FET with a channel length ranging from 10 to 20 nm. Yttrium doping (Y-doping) was applied at the top layer of the few-layered InSe to improve the contact between the 2D channel and the electrode, which induces a phase transition from semiconductor to semimetal. The Y-doped InSe and the top layer of pristine InSe have no Fermi Level Pinning effect, so the ohmic contact is formed, having a small resistance of 64 Ω μm. An ultrathin high-*k* dielectric material, HfO_2,_ with an EOT of 2.6 nm, was utilized as the gate oxide. It is quite challenging to grow an ultrathin high-*k* dielectric layer on the dangling-bond-free surface of 2D materials. The best on-state current and transconductance are achieved for the triple-layer (TL) InSe FET with *L*_g_ = 20 nm, at 1.20 and 1.43 mA μm^−1^ and 6.0 and 7.2 mS μm^−1^ at *V*_dd_ of 0.5 and 0.7 V, respectively. The triple-layer 2D InSe *I*_on_ attains a theoretically predicted *I*_on_ of 1.5 mA μm^−1^ at *L*_g_ = 7 nm.^12^ The significantly high on-state current is due to reduced carrier scattering because of small surface roughness and the dangling-bond-free surface. On the other hand, the high on-state current in multilayer devices is attributed to the availability of a large density of states. Notably, the on-state current of 2D InSe FETs decreases monotonically from TL to ML. The poor performance of ML 2D InSe FETs is ascribed to direct Y-doping on the ML InSe to form electrodes. Covalent interactions occur in the lateral direction between Y–InSe and the semiconductor InSe, creating metal-induced gap states and resulting in a Schottky barrier. Another reason for the poor performance of low-current ML 2D InSe is the structural instability. An improved approach is utilizing BL InSe as electrodes, with doping applied only on the top layer. This method facilitates the formation of an Ohmic contact between the doped and undoped InSe layers.

Our theoretical simulation study predicts that, by realizing an ultrathin high-*k* dielectric of 1.5 nm and by realizing ohmic contact electrodes, ML and BL n-type β-InSe FETs outperform at *I*_on_ values of 1236 and 648 μA μm^−1^, respectively. The thicker channel experiences a decline in the degradation of the electrostatic control exerted by the gate.

## 5 Conclusion

In this work, we studied the ballistic limit of sub-3 nm ML and BL DG n-type β-InSe FETs by employing *ab initio* quantum transport simulations. The optimized ML n-type β-InSe FET was used at *L*_g_ = 2 and 3 nm to explore the performance of devices for high *I*_on_ of 1236 and 1291 μA μm^−1^, respectively. Thus, to further study few-layer n-type β-InSe FETs, the best device configuration was selected with *L*_g_ = 2 nm and an optimal *L*_UL_ of 1.5 nm to keep a *L*_ch_ of 5 nm, and employing a high-*k* dielectric HfO_2_ gate dielectric with a thickness of 1.5 nm. It is predicted that ML and BL n-type β-InSe FETs can easily fulfill the HP ITRS device requirements. Other crucial figures of merit, such as *τ*, PDP, and EDP, for ML and BL n-type β-InSe FETs are well matched with HP ITRS requirements of the 2013 version for the 2028 target. Thus, ML and BL n-type β-InSe FETs outperform several other 2D-material FETs, demonstrating strong potential for future nanoelectronics applications.

## Conflicts of interest

There are no conflicts to declare.

## Data Availability

The data that support the findings of this study are available from the corresponding author upon reasonable request.
